# The influence of organizational agility on nurses` career planning: nurses` grit as a mediating factor

**DOI:** 10.1186/s12912-024-02303-2

**Published:** 2024-09-26

**Authors:** Amal Diab Ghanem Atalla, Mohamed Saad Saleh Ali, Ayman Mohamed El-Ashry, Wafaa Hassan Mostafa

**Affiliations:** 1 Department of Nursing Administration, Faculty of Nursing, Alexandria University, Alexandria, Egypt; 2 Department of psychiatric and mental health nursing, Faculty of Nursing, Alexandria University, Alexandria, Egypt; 3 Department of Nursing Administration, Faculty of Nursing, Damanhour University, El-Behira, Egypt; 4 Assistant professor at nursing department, college of applied medical sciences, Jouf university, Al-Qurayyat, Saudi Arabia

**Keywords:** Organizational agility, Nurses, Career planning, Grit, Mediating factor

## Abstract

**Background:**

Organizational agility is the cornerstone of the complex dynamics for the success of healthcare organizations and the sustenance of nurses’ career planning and professional development.

**Aim:**

Assess the organizational agility and the extent of nurses’ career planning. It also sought to investigate the association between the two and the intermediating function that nurses` grit plays in this association.

**Design:**

A cross-sectional descriptive design following STROBE guidelines was carried out.

**Methods and tools:**

Data were collected from 300 nurses who made up the entire population sample that we used (a judgmental non-probability sampling technique) working across all in-patient care units at Itay El-Baroud General Hospital, in El Behaira governorate, using structured questionnaires as follows: the organizational agility questionnaire, the career planning scale, and the grit scale. The sociodemographic characteristics of the studied nurses were also collected. Statistical analyses were used to analyze the data, including ANOVA, Pearson correlation, and SEM, to determine whether career development may act as a mediator between organizational agility and the nurses’ career planning.

**Results:**

Rendering the findings of this study, more than half of nurses have a high perception regarding organizational agility and career planning. In contrast, the majority of them have a moderate perception regarding grit. In addition, there is a statistically high significant relationship between organizational agility and career planning. Also, there is a statistically high significant relationship between organizational agility and grit. There is a statistically high significant relationship between career planning and grit.

**Conclusion:**

The statistically substantial correlations among grit, career planning, and organizational agility demonstrate how interrelated they are. This implies that encouraging organizational flexibility and effective career planning techniques may have a good impact on nurses’ resilience, which would ultimately result in a workforce of nurses who are more resilient and engaged. Additional investigation into the precise mechanisms behind these associations may yield important information for improving nurse wellbeing and retention.

**Nursing implications:**

According to this study, putting organizational agility and career planning first can have a good impact on nurses’ grit, which will make them more resilient and engaged workers. To encourage flexibility and growth among nurses, healthcare institutions should make training investments, provide clear career tracks, and support work-life balance. More investigation into the precise mechanisms underlying these connections may yield insightful information that improves nurse well-being and retention. The importance of organizational agility in creating a supportive work environment for nurses’ career growth should be acknowledged by healthcare companies. This can lead to increased job satisfaction and lower turnover.

## Background

Many healthcare organizations revise their strategic priorities and emphasize adapting to changes in the work setting and quick response to patient needs, starting to demonstrate the necessary competencies to adapt to these changes in real time [1]. The concept of organizational agility was offered as a means of ensuring the existence, success, and survival of healthcare organizations. Organizational agility is defined as “the ability to respond and adapt quickly, creatively and successfully to the unexpected changes brought by opportunities and threats that are likely to occur in the internal and external environment of an organization and to achieve high standards of performance, and deliver quality services“ [2]. The Global Strategic Directions for Nursing and Midwifery (SDNM) 2021–2025 presents evidence-based practices and an interrelated set of policy priorities that can help countries ensure and facilitate the development of sustainable organizational agility by improving nurses’ career planning and strengthening nurses’ grit [3].

The “State of the World’s Nursing 2020” report from the World Health Organization, which promotes funding for nursing leadership, employment, and education, has a major impact on global health policy. The ultimate goals of this report are to strengthen nursing and enhance healthcare access globally by promoting essential competencies, firming up the Nursing profession, and shaping nursing care paradigms [4]. Also, the “Global Strategic Directions for Nursing and Midwifery 2021–2025” published by the World Health Organization provides a road map for advancing midwifery and nursing internationally. This statement aims to improve healthcare outcomes and access globally by actively influencing global health policy, developing competency development, and advocating for the progress of the nursing profession [3]. Nurses’ active participation in establishing international health policy is demonstrated by the World Health Organization’s “Nursing and Midwifery” (2022) resource. The ultimate goal of this statement is to improve healthcare access and outcomes globally by influencing the creation of nursing care models, promoting the development of critical nursing and midwifery abilities, and advocating for the promotion of these professions [5].

In addition, the crucial role that nurses play in fortifying and reconstructing healthcare systems is highlighted in the International Council of Nurses’ “Recover to Rebuild: Investing in the Nursing Workforce for Health System Effectiveness” (2023) report. This report advocates for more investment in nursing education, jobs, and leadership positions, which has a direct impact on global health policy. In the end, it seeks to enhance healthcare outcomes and accessibility globally by advocating for the improvement of the nursing profession and encouraging the development of critical nursing competencies [6].

## Literature review

### Organizational agility (OA)

Organizational agility was identified by four main dimensions: flexibility, responsiveness, competencies, and sensing capability. *Flexibility* is” the ability to carry out any process in a different way and any situation to achieve the organization’s goals”. *Responsiveness* is “the ability to identify and respond reactively and constructively to any changes that may appear in an environment”. *Competencies* are “the extensive set of abilities that provide productivity, efficiency, and effectiveness of activities towards the aims and goals of the organization”. *Sensing capability* is “the ability of an organization to inspect, interact and deal with events and any changes that may affect the organization” [4].

Strengthening the organizational agility of the hospital can lead to better serving patients’ needs, introducing new services, and increasing competitiveness [7]. It also improves organizational capabilities, decreases human error, maintains high quality and low cost, enhances job satisfaction, and achieves organizational goals [8]. The presence of organizational agility can decrease challenges facing nursing staff, encouraging health organizations and nurses to share the responsibility of planning their careers. Also, organizations can strengthen nurses` career planning, providing them with growing opportunities and building a successful career plan [9].

### Career planning (CP)

The recent and rapid changes in health led to changes in the nurses` career choices. Planning a career is an ongoing, iterative process that includes getting to know oneself, desires, and expectations, setting career goals and the means to fulfill them, and revising professionals` skills, capabilities, and opportunities [1]. It also refers to the time, direction, and plan of action the nurse sets to achieve their career goals and it becomes a part of the repertoire of skills and experiences that enables nurses to develop their profession [10].

Career planning includes six domains to achieve career development and career maturity as follows: *Knowledge of the world of work* means “understanding how the global economy will affect the career and taking advantage of new technology and new career opportunities”. *Self-knowledge* refers to “an ability to learn about oneself as a result of the information obtained and furnished through the evaluation experience; interests and career aspirations” *Knowledge of occupation* involves “exploring and talking to others about interesting occupations”. *Career decision-making* includes “collecting the needed information, weighing the costs and benefits of the choices and making final decisions that fit the personal characteristics and overall life goals”. *Career planning* means” developing both short- and long-term goals for the career development within a specific timeline for accomplishing these goals”. *Career implementation* refers to “knowing how to implement occupational decisions once making them, knowing how to look for jobs in both the visible and hidden job markets” [11].

Career planning can effectively solve professional growth difficulties, improve career maturity, and increase the sense of career identity [10]. It can help nurses develop their healthcare professionals, achieve organizational goals, and provide strong support for them [12, 13]. Lack of career planning affects nursing staff specialization, turnover rate, and job satisfaction [14–16]. Career planning is a main for the gritty nurses who are working energetically towards challenges, support effort, and interest toward goals, where consistency of interests and disposition to persist in challenging times is positively related to nurses’ capability to create their career goals and plans. Additionally, grit positively influences career planning developed to accomplish peak career goals [17].

### Nurses’ grit (NG)

Grit is attracting attention as a significant factor that can successfully enhance the nursing profession. It is defined as perseverance and passion for long-term goals; moreover, it is the driving force for overcoming failure, adversity, and frustration in achieving long-term goals [13]. The grit has two main dimensions: perseverance of effort and consistency of interest. *The perseverance of effort* refers to the degree to which one perseveres and continuously strives through working extremely hard, overcoming obstacles and failures while sustaining intense effort to achieve that goal. Further, *consistency of interest* refers to pursuing a goal while maintaining interest in that goal for a long time as many years [18].

Grittier nurses tend to seek understanding to continually move forward to adopt various strategies to achieve their goals [19]. They also leverage the power of teamwork to persevere in achieving collective goals of improving patient outcomes, building their professional knowledge and skills, and showing greater positive behaviors where nurses actively seek out new resources and innovative solutions [20]. Grit plays an essential role in nurses’ work ability, job satisfaction, and overall performance, which may also reduce staff turnover rate and increase retention [21]. Professionals with a high level of grit continue to work hard while changing their behaviors as needed to achieve the goal [22]. Grit emphasizes perseverance to overcome encounters and uniformity of interest essential to accomplish long-term goals [23].

### Theoretical framework

**Fig. 1:**
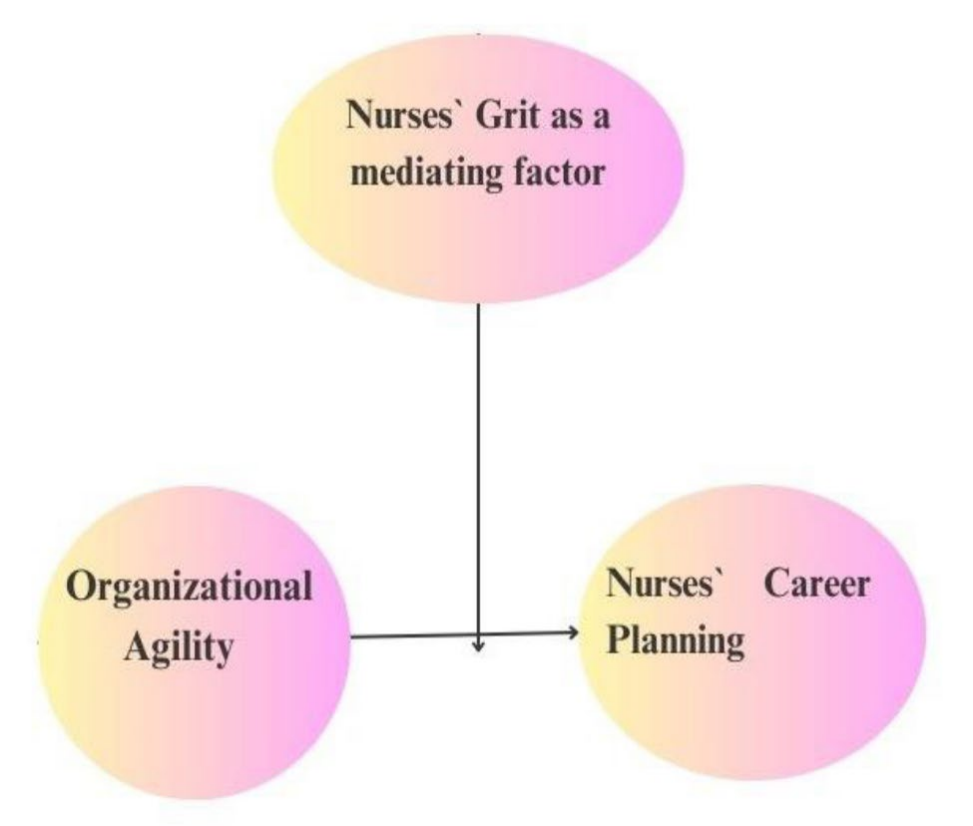
The researchers’ proposed conceptual framework of the study

The enlightenment provided by career theory explains how people make choices, establish desires, and perform at varying levels about their academic and professional goals. Systems for understanding how various elements interact to affect career prospects and advancement throughout a person’s life are provided by career theories. Dos Santos (2021) [24] showed that Social Cognitive Career Theory (SCCT) is a holistic theory derived from the general social theory of Bandura (1986) [25]. SCCT is a holistic theory that focuses on the part that specific cognitive traits play in vocations. This highlights the intricate relationships that exist between people, their environments, and their behaviors. It is argued that social context and self-efficacy affect people’s conduct. According to SCCT, individuals possess the capacity for some level of self-direction or organization, but they also face several challenges that can either bolster or undermine personal organization. From the aforementioned conceptualizations, we planned a conceptual model for this study (Fig. 1). Assumed that organizational agility is the independent variable, nurses` career planning is the dependent variable, and nurses` grit acts as a mediating role, the following conceptual framework is postulated:

### Significance of the study

This study holds significant relevance for the nursing profession in Egypt. It addresses a notable and crucial gap in the existing literature. This is significant as it provides insights and evidence-based knowledge tailored to the unique challenges and dynamics of the Egyptian healthcare system. Organizational agility is crucial in the healthcare sector, where nurses face dynamic and evolving challenges.

Using an integrative approach, this research incorporates organizational agility and nurses’ grit as important characteristics rather than just looking at organizational or individual elements. Furthermore, the examination of grit as a mediating factor between organizational agility and nurses’ career planning has revealed a novel mediating mechanism that sheds light on the psychological processes at play. The study’s contextual focus, which resulted from its execution in the unique nursing setting, further advances the field’s comprehension of career growth in this vital healthcare profession.

One of this study’s main contributions and novelties is that most earlier research has concentrated on organizational or individual issues alone. The relationship between organizational agility and how it affects nurses’ career planning is examined in this study. Furthermore, because the research’s conclusions can assist healthcare organizations in creating practices, policies, and strategies that support organizational agility and make better use of nurses’ tenacity in career planning and retention, the study has immense importance and influence within the scientific community. This study adds to our understanding of the variables affecting nurses’ career paths, especially in light of the quickly evolving healthcare landscape.

With the status of the nursing workforce today, it is concerning that there is a dearth of information regarding the impact of organizational agility and grit on career planning among nurses. Examining how this demanding workplace impacts nurses’ career planning is crucial now more than ever, given the extreme shortage of highly qualified nurses and the high cost of replacing an experienced nurse. A complete analysis of the study was conducted to analyze the relationship between organizational agility and career planning among nurses and estimate the potential direct and moderating consequences of grit in Egypt, close to the most noteworthy research information and literature evaluation. Additionally, we expect that by illuminating the complex relationship between career planning and organizational agility among nurses. The study aimed to fill up a knowledge vacuum in this regard. Therefore, this study aimed to investigate how nurses’ grit mediated the relationship between organizational agility and career planning among nurses.

### The research questions are

- What are the levels of organizational agility, career planning, and grit among staff nurses?
- Is there a relationship between organizational agility, career planning, and grit among staff nurses?
- Is there a mediating effect for nurses’ grit between organizational agility and nurses’ career planning among staff nurses?

### Study design

A cross-sectional descriptive correlational research design was implemented according to STROBE guidelines in an Egyptian hospital.

### Setting

The study was conducted at Itay El Baroud General Hospital, which is affiliated with the Ministry of Health and Population (MOHP), with bed capacity (*n* = 220) beds. It included all medical and surgical inpatient care units and Intensive Care Units (ICUs) (*n* = 16), namely: (1) medical units (*n* = 8), namely: general medical (Male and Female), obstetric, pediatric, poison, orthopedic, neurosurgical and dialysis units; (2) surgical units (*n* = 3), as follows: general surgical (Male and Female) and operation units; (3) ICUs (*n* = 5), as: general, neonatal, pediatric, coronary care and emergency units. This hospital started serious steps to fulfill the requirements of the General Authority for Health Accreditation and Regulation (GAHAR) regarding patient safety. This hospital is one of the largest hospitals in Egypt and the Middle East, it serves a sizable population across a broad catchment area. It serves as a tertiary referral center for numerous hospitals around the country as well as frequently for those in neighboring nations. Accordingly, better patient outcomes and higher-quality healthcare services can result from enhanced nurse career planning and retention, which has positive social consequences for the entire community.

### Sampling

This is a convenient sampling (a non-probability sampling technique where researchers select participants based on their easy accessibility and availability). All target populations of nurses were included in the study. The study (*n* = 300) comprised nurses who had worked in the aforementioned units for a minimum of one year to familiarize them with the hospital system as well as administrative rules, policies, and regulations. Additionally, the nurses needed to be present when the data was being collected. The following criteria were fulfilled by nurses who were selected to take part in the study: (1) They had to be employed in settings that were pre-selected; (2) They had to have spent a minimum of one year in the working unit; and (3) They had to be offering nursing care to patients directly.

### Study tools

#### Sociodemographic characteristics section

The study participants’ years of work unit, gender, age, education, and nursing experience were among the items the researchers questioned.

### Organizational agility questionnaire (OAQ)

It was developed by Shajrawi (2018) [26] and was adapted by the researcher to assess organizational agility. It includes 24 items grouped into four main dimensions namely; (1) flexibility (8 items); (2) responsiveness (5 items); (3) competencies (5 items); and (4) sensing Capability (6 items). Responses to the questionnaire items were measured on a five-point Likert rating scale ranging from (1) strongly disagree to (5) strongly agree. The overall mean score level of the Organizational Agility Questionnaire (OAQ) ranges from 24 to 120. This scale’s Cronbach’s alpha score was 0.922 [26]. In the present study, Cronbach’s alpha was 0.95.

### Career planning scale (CPS)

This tool was developed by Liptak (2008) [11] to measure skills required for career planning. The scale consists of 48 items divided into six main dimensions as follows: knowledge of the world of work, self-knowledge, knowledge of occupations, career decision-making, career planning, and finally, career implementation, each dimension contains 8 items. The responses were measured on 5- a 5-point Likert scale ranging from strongly agree (5) to strongly disagree (1). The overall score ranged from 48 to 240. The score ranged from 48 to 111. The construct validity was verified (Internal Correlations Matrix between the dimensions of the scale ranged from 0.54 to 0.74 [11]. The Cronbach’s alpha score was between 0.75 and 0.86 [11]. In the existing study, Cronbach’s alpha was determined as 0.85.

### The grit scale

Duckworth et al. (2007) [27] created and validated this Scale. The 12 items on the scale are broken down into two categories: perseverance of effort and consistency of interests. As a result, Duckworth et al. (2007) [27] suggest using the overall scores to measure grit. They discovered that the two elements together were more predictive of outcomes than either factor alone. According to Duckworth et al. (2007) [27], the 12-item Grit Scale “demonstrated high internal consistency for the overall scale (Cronbach’s α = .85). Similar to this, Eskreis-Winkler et al. (2014) [28] reported that the 12-item grit scale has good internal consistency in two research; the reliability coefficients for the first study are α = .77 and the second study is α = .79.

The six items in the Perseverance of Effort factor include, the respondent rates each item on a five-point Likert scale, ranging from “1-not at all like me to 5 very much like me.” As an illustration, consider the reverse-scored six items related to the Consistency of Interest factor [27]. In the current study, Cronbach’s alpha was 0.82.

### Tools validity

The three instruments underwent modifications, an Arabic translation, and an English translation. Seven experts were provided with the resources to analyze, assess, and provide comments on item clarity, question types, and content validity. These experts were two lecturers and five professors from the Nursing Administration Department. Their recommendations were taken into account to guarantee accuracy and shield the study from danger. To ensure accuracy, a confirmatory factor analysis was performed for the grit scale, career planning scale, and organizational agility questionnaire. The earliest tools used to assess sample adequacy were the Bartlett Test of Sphericity and the Kaiser-Meyer-Olkin (KMO). It is necessary to have a minimum KMO value of 0.60 and a Bartlett Test of Sphericity significance level of 0.05.

The organizational agility scale had a value of 0.899 (P 0.000), the career planning scale had a value of 0.918 (P 0.000), and the grit scale had a value of 0.931 (P 0.000), according to the data. Factor loadings for every concept that was investigated in this study were higher than the recommended threshold of 0.70 [29], indicating that the scales’ construct validity is supported. Furthermore, the average variance extracted (AVE) values for all research variable dimensions show that convergent validity is satisfied [30]. Convergent validity was assessed using each construct’s average variance extracted (AVE) values. AVE values greater than 0.50 indicate that the construct explains most of the variance, confirming convergent validity. Discriminant validity was assessed by comparing the AVE values to the squared correlations between constructs. All AVE values were higher than the squared correlations, indicating that discriminant validity is satisfied. Thus, both discriminant and convergent validity were confirmed for the scales used in this study.

### Ethical considerations

The Damanhour University Research Ethics Committee, which is part of the College of Nursing, approved the study procedure. The study’s purpose was communicated to nurses before they gave their written consent. Each questionnaire was given a code number to protect confidentiality and identity. As promised to nurses, data use was limited to research. The right to withdraw from the study has been verified.

### Pilot study and reliability

The pilot study was approved by 10% of the nurses (*n* = 30) to protect the practicality and simplicity of the goods and to identify potential barriers and issues during data collection. There was nothing that needed to be altered. The study did not include those who took part in the pilot study. The questionnaires were checked by the researchers for accuracy and inclusivity. The Cronbach’s alpha coefficient test was used to evaluate the internal consistency of the items to gauge the dependability of research instruments.

### Overcame the problem of common method biases

The authors addressed the issue of common method bias (CMB) through a combination of design and procedural techniques and statistical controls. Procedurally, they assured participants anonymity and confidentiality to reduce social desirability bias and encourage honest responses. They also separated different measures within the questionnaire, providing clear instructions and distinct response formats to minimize the tendency of respondents to provide consistent answers across items. Statistically, the authors conducted Harman’s single-factor test, which indicated that no single factor accounted for most of the variance, suggesting that CMB was not a significant concern. Additionally, confirmatory factor analysis (CFA) was used to validate the measurement model and ensure that the constructs were distinct and not overly correlated, reducing the likelihood of CMB. The authors also conducted a pilot study with 10% of the sample to refine the questionnaires, ensuring clarity and reducing ambiguity, which can contribute to CMB. By combining these approaches, the authors effectively mitigated the potential impact of common method bias on their study’s results.

### Data collection

Personalized copies of the study questionnaires were given to the participants. The researchers gave a hand-delivered questionnaire to each nurse, and then collected the completed forms later. After explaining the goal of the study to each nurse individually for two minutes, the nurses were asked to return it to the researcher. These scales were completed in front of the researcher to ensure the respondents’ objectivity, the integrity of their thoughts, and the completion of all questions. Because they were connected to certain working units, it was easy to monitor the delivery and gather to guarantee the highest response rate. In exchange for their participation, participants received little snacks. The questions should take 15 to 20 min to complete. Three months passed between March 2024 and June 2024 to gather the information. All of the nurses’ questions were answered, and explanations were given.

### Data analysis

The data was analyzed using IBM SPSS AMOS (Version 23) and IBM SPSS Statistics (Version 23). The demographic features of the participants were described using frequency and percentage. The three main study variables—organizational agility, career planning, and nurses’ grit—were defined using means and standard deviations. An independent sample t-test and a one-way analysis of variance were used to identify changes in the research variable based on demographic characteristics. Pearson’s correlation analysis was utilized to determine the association between the essential research variables. To determine the direct impact of organizational agility on career planning, regression analyses were employed. Using a structural equation model, the indirect impact of organizational agility on nurses’ grit in career planning was examined. The study employed Cronbach’s alpha and composite reliability (CR) to verify the validity of the scale items. Furthermore, multiple confirmatory factor analyses were conducted to ensure the precision of the study components.

## Results

According to the study’s findings, 77.7% of nurses are under 30 years old, and 80.7% of nurses are female. In addition, 51.7% of nurses have married lives. Additionally, 9.3%, 10.7%, and 37.0% work in critical care, surgery, and internal medicine. A bachelor of science in nursing degree is held by 64.7% of nurses. The mean duration of employment as a nurse is 4.4 ± 4.9 years. Furthermore, 73.7% of nurses have spent less than five years working in their departments. Additionally, 77.7%, 71.7%, and 72.3% of nurses reported not having taken grit, career planning, or organizational agility training courses. Additionally, 86.3% and 89.0% of respondents desire to take training programs in grit, career planning, and organizational agility (Table 1).

**Table 1:** Distribution of the studied nurses according to demographic data (*N* = 300)

Demographic characteristics	No.	%
**Sex**		
Male	58	19.3
Female	242	80.7
**Age (years)**		
< 30	233	77.7
30 –<40	54	18.0
40 –<50	13	4.3
Mean ± SD	20.8 ± 4.0	
**Marital status**		
Single	136	45.3
Married	155	51.7
Divorced	6	2.0
Widowed	3	1.0
**Unit**		
Internal medicine	28	9.3
Surgical	32	10.7
Critical	111	37.0
Other	129	43.0
**Educational level**		
Technical Nursing Institute	58	19.3
Bachelor science in nursing	194	64.7
Diploma	20	6.7
Fellowship	3	1.0
Masters	12	4.0
PhD	13	4.3
**Experience year of nursing**		
< 5	169	56.3
5 -<10	80	26.7
10 -<15	40	13.3
15 -<20	6	2.0
≤ 20	5	1.7
Mean ± SD	4.4 ± 4.9	
**Experience years of unit**		
< 5	221	73.7
5 -<10	46	15.3
10 -<15	22	7.3
15 -<20	6	2.0
≤ 20	5	1.7
Mean ± SD	3.3 ± 4.3	
**Did you attend training courses about Organizational agility**		
No	233	77.7
Yes	67	22.3
**Do you want to attend training course about Organizational agility**		
No	41	13.7
Yes	259	86.3
**Did you attend training courses about Career planning**		
No	215	71.7
Yes	85	28.3
**Do you want to attend training course about Career planning**		
No	33	11.0
Yes	267	89.0
**Did you attend training courses about Grit**		
No	217	72.3
Yes	83	27.7
**Do you want to attend training course about Grit**		
No	33	11.0
Yes	267	89.0

### Perceived level of organizational agility, career planning, and grit among nurses

52.7% and 58.7% respectively of the studied nurses have positive opinions of organizational agility and career planning, respectively. In addition, the majority of nurses (89.7%) view grit as moderate. Ultimately, the mean score for career planning was the highest (Mean ± SD = 177.03 ± 27.78) (Table 2).

**Table 2:** Distribution of the studied nurses according to their levels and mean percent score of Organizational agility questionnaire, Career planning scale, and Grit Scale (*N* = 300)

	Low		Moderate		High		Total score	Mean percent score
	No.	%	No.	%	No.	%	Mean ± SD	Mean ± SD
Flexibility	4	1.3	118	39.3	178	59.3	30.12 ± 4.76	69.14 ± 14.86
Responsiveness	21	7.0	138	46.0	141	47.0	17.70 ± 3.85	63.50 ± 19.23
Competencies	26	8.7	132	44.0	142	47.3	17.64 ± 4.15	63.18 ± 20.73
Sensing Capability	13	4.3	114	38.0	173	57.7	21.66 ± 4.49	65.25 ± 18.69
**Organizational agility questionnaire**	6	2.0	136	45.3	158	52.7	87.12 ± 15.55	65.75 ± 16.20
Knowledge of the World of Work	6	2.0	135	45.0	159	53.0	29.68 ± 4.81	67.75 ± 15.04
Self-Knowledge	7	2.3	72	24.0	221	73.7	31.31 ± 4.99	72.84 ± 15.60
Knowledge of Occupations	14	4.7	116	38.7	170	56.7	29.28 ± 5.37	66.50 ± 16.78
Career Decision-Making	16	5.3	113	37.7	171	57.0	29.31 ± 5.71	66.58 ± 17.85
career Planning	18	6.0	144	48.0	138	46.0	28.19 ± 5.67	63.08 ± 17.73
Career Implementation	12	4.0	119	39.7	169	56.3	29.27 ± 5.58	66.47 ± 17.42
**Career planning scale**	6	2.0	118	39.3	176	58.7	177.03 ± 27.78	67.20 ± 14.47
Perseverance of Effort	4	1.3	71	23.7	225	75.0	23.78 ± 4.19	74.10 ± 17.47
Consistency of Interest dimension	156	52.0	137	45.7	7	2.3	13.65 ± 4.16	31.86 ± 17.33
**Grit Scale**	2	0.7	269	89.7	29	9.7	37.43 ± 4.25	52.98 ± 8.85

SD: Standard deviation Low (< 33.3%) Moderate (33.3 – <66.6%) High (≥ 66.6%)

### Relationship among organizational agility, career planning, and grit among nurses

The results also, indicate that all correlations were statistically significant at *p* = 0.05, according to the correlation matrix of the links between organizational agility, career planning, and nurses’ grit based on a sample size of 300. The grit of nurses and career planning had a positive correlation (*r* = 0.116) with organizational agility (*r* = 0.149). This shows that increases in career planning and nurses’ grit are linked to increases in organizational agility. Additionally, there was a strong correlation between career planning and nurses’ grit (*r* = 0.166), suggesting that they tend to advance together (Table 3).

**Table 3:** Correlation between the studied variables (*N* = 300)

		Organization agility questionnaire	Career Planning
**Career Planning**	**R**	0.149*	
	**P**	0.010*	
**Overall Grit**	**R**	0.116*	0.166*
	**P**	0.045*	0.004*

r: Pearson coefficient

*: Statistically significant at *p* ≤ 0.05

### Standardized regression coefficient weights among organizational agility, career planning, and grit with the mediating role of nurses’ grit

**Fig. 2:**
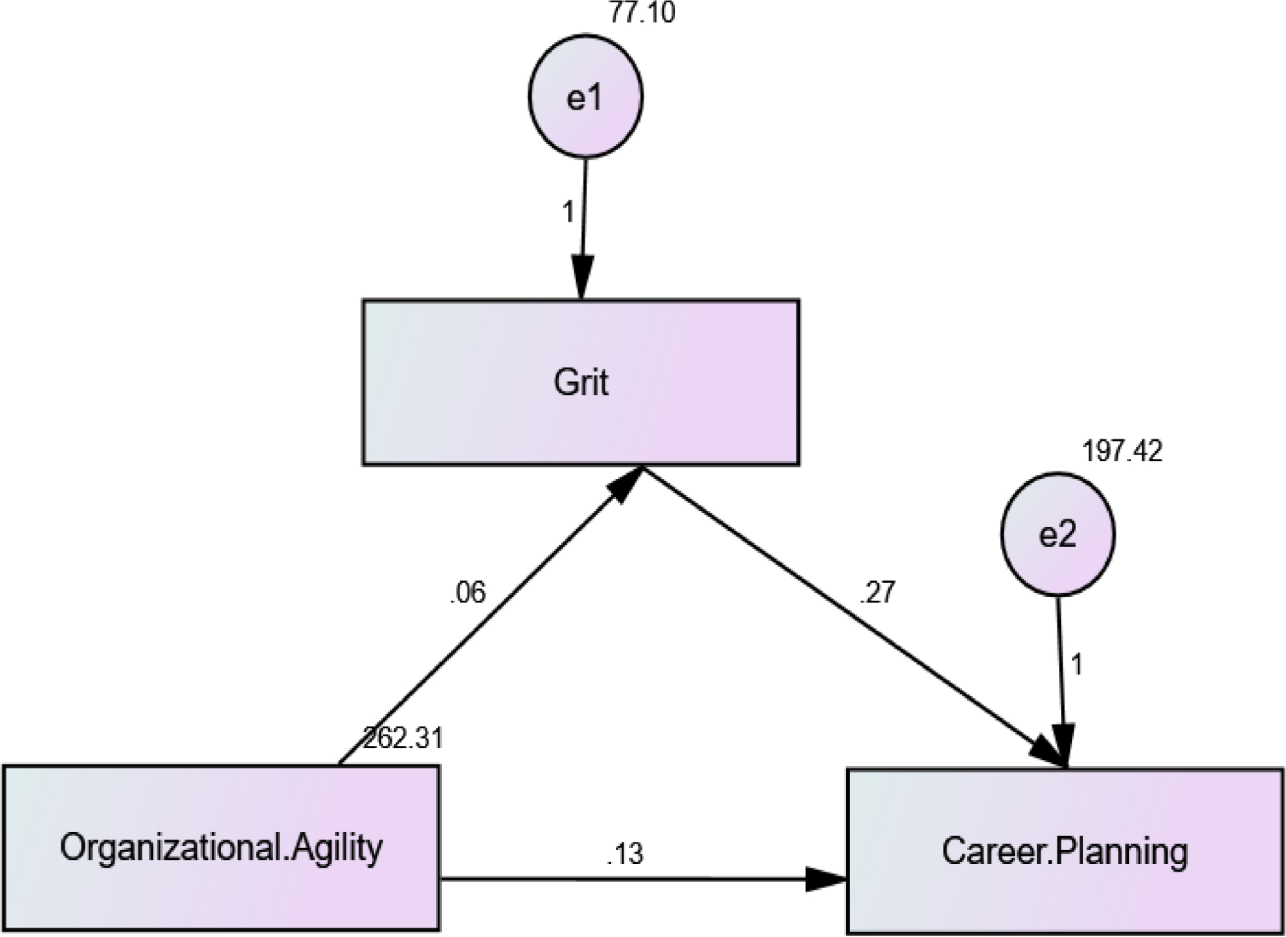
: Path analysis of the direct and indirect effect of Organizational Agility on Nurses’ Career Planning: Nurses’ grit as a mediator

The study outlines the consequences of career planning, organizational agility, and nurses’ grit, both directly and indirectly. Nurses’ grit is directly impacted by organizational agility (an independent variable) (mediator). The path coefficient of 0.115 (p 0.046) illustrates this. Career planning (dependent variable) is directly impacted by nurses’ grit (mediator). The path coefficient of 0.162 (*p* = 0.004) serves as an indicator of this. Career planning (dependent variable) is directly impacted by organizational agility (independent variable). The path coefficient of 0.143 (p 0.012) represents this. Multiplying the coefficients of both direct effects yields the indirect effect of organizational agility on career planning through nurses’ grit (0.115 * 0.162 = 0.709). Concerning the mediating role of nurses’ grit, we would anticipate a 0.709 unit rise in career planning for every unit increase in organizational agility. The model appears to match the data well based on the model fit parameters (CFI = 1.000, IFI = 1.000, RMSEA = 0.138). The disagreement between the data and the model is measured by the Chi-square value per degree of freedom (X2/df = 6.662/3). A better fit is indicated by a smaller value. There is a perfect fit when both the incremental fit index (IFI) and comparative fit index (CFI) equal 1.000. With a Root Mean Square Error of Approximation (RMSEA) of 0.138, which is less than the generally recognized cutoff of.08, the approximation error is fair. All of these indicators point to a good representation of the observed data by the model (Table 4 and Fig. 2**)**.

**Table 4:** The direct and indirect effect of Organizational Agility on Nurses’ Career Planning: Nurses’ grit as a mediator

			Direct effect	Indirect effect	Estimate	S.E.	C.*R*.	*P*
**Grit**	**<---**	**Organizational Agility**	0.063	0.0	0.115	0.031	1.998*	0.046*
**Career Planning**	**<---**	**Organizational Agility**	0.127	0.057	0.143	0.051	2.524*	0.012*
**Career Planning**	**<---**	**Grit**	0.265	0.0	0.162	0.093	2.865*	0.004*

Model fit parameters CFI; IFI; RMSEA (1.000; 1.000; 0.138)

Model χ^2^/df. 6.662/3 *p* ≤ 0.001

CFI: Comparative Fit Index, IFI: Incremental Fit Index, RMSEA: Root Mean Square Error of Approximation

## Discussion

Healthcare organizations can ensure their existence, success, and survival by using organizational agility [2]. The organization must be agile and implement corporate reorganization to respond to competition and adapt to changes in the internal and external environment. This includes restructuring strategies to meet high-performance standards and provide quality patient care services [1, 2]. Agile healthcare organizations encourage career planning techniques that provide the healthcare organization a competitive edge by producing gritty nurses who take on obstacles head-on and encourage effort and interest in reaching career planning objectives [17].

### Perceived level of organizational agility, career planning, and grit among nurses

The study outcomes confirmed that most nurses had high perceptions of organizational agility. This could be explained by the nurses’ perception that the hospital adapts well to changes in patient care services and demands, handles patient complaints promptly, effectively gathers and uses market data to improve the quality of patient care services, and routinely holds interdepartmental meetings in response to outside changes.

The findings of this study are in line with those of (Ali, et al., 2022) [31], who found that slightly more than half of staff nurses reported high levels of organizational agility and career aspirations. The results of this study also support the findings of (Athamneh and Jais, 2023) [32], (Karafakıoglu and Findikli, 2024) [33], and (Akkaya, et al., 2024) [34] who reported that most nursing staff have a high degree of organizational agility. The results of this study also align with those of Mohammed et al. (2024) [35], who found that staff nurses reported increased job agility and career aspirations.

This study also discovered that most nurses have high perceptions of career planning. This might be related to the fact that nurses assumed truthfully about the opportunities that were open to them, created a plan for lifelong learning and the continuous acquisition of new skills, recognizing their interests, talents, and motivators, and gathered enough data to make wise decisions about their careers, created short- and long-term career goals. The results of this study are consistent with those of other studies (Salleh et al., 2020; Yan et al., 2020; Mohamed et al., 2021; Ali et al., 2022; Fitzpatrick et al., 2023) [12, 13, 31, 36, 37] that found that nurses had high levels of career planning. The results of this study also align with those of Mohammed et al. (2024) [35], who found that nurses reported high levels of career planning. Furthermore, the study’s findings are in line with those of Zhang et al. (2021) and Zeng et al. (2023) [38, 39], who reported that nursing students had high levels of career planning. Also, the current study’s findings are consistent with those of El-Bahnasawy et al. (2021) [14], Abou Hashish and Bajbeir (2022) [40], Xie et al. (2023) [41], and Landoll et al. (2023) [42] who found that nursing students exhibited high levels of career planning knowledge.

Furthermore, the study results revealed that most nurses have moderate perceptions of grit. This could be explained by the notion that nurses are perceived as hard workers and diligent and that they can overcome obstacles to overcome a significant challenge, stay focused and interested in a project or idea for an extended time, set long-term goals that require years of work and persevere to achieve them, and be interested in trying new things every few months. The findings of this study are consistent with those of (Lynda, 2019) [18], the findings of Terry and Peck (2020) [43] and Shin (2022) [44]. (Cindy and Hope, 2019) [45], and (Landoll, et al., 2023) [42] noted that nursing students had a modest level of grit. Additionally, the results of this study are consistent with those of Cherian and Kumari (2021) [46], who found that nurses had a moderate level of grit. Additionally, the current study’s findings support those of Yang et al. (2022) [47] and Lofton (2022) [48], who found that nursing staff showed a moderate degree of grit. Furthermore, the results of this study are consistent with those of Liu et al. (2024) [49] and Terry et al. (2024) [50], who reported that nurses exhibited a moderate level of grit.

### Relationship among organizational agility, career planning, and grit among nurses

The results of this study disclosed that career planning was correlated with organizational agility (*r* = 0.149). This finding implies that organizational agility is one of the leading indicators of career planning. This can be explained by the fact that agile healthcare organizations can respond and adapt swiftly, creatively, and successfully to the unforeseen changes brought about by opportunities and threats in the internal and external environment of an organization. As a result, organizational agility contributes to better career planning in a way that helps nurses advance their careers and meet their career planning objectives.

This finding is consistent with a study by Clauss et al. (2021) [1] and Mahmoud et al. (2022) [2], who demonstrated that organizational agility enables healthcare organizations to successfully respond and adapt to unforeseen changes brought about by opportunities and threats that are likely to arise in the organization’s internal and external environment. Furthermore, this outcome is consistent with the findings of (Dizari and Garoosi, 2019) [8] ; (Hussein et al., 2022) [9], and (Basiony & Ibrahim, 2023) [7] who stated that organizational agility helps nurses fulfill their career planning objectives and achieve the success they desire inside the organization.

### Standardized regression coefficient weights among organizational agility, career planning, and grit with the mediating role of nurses’ grit

Furthermore, the results of this study displayed nurses’ grit as a mediator between organizational agility and career planning. Organizational agility (independent variable) directly affects nurses’ grit (mediator). This is represented by the path coefficient of 0.115 (p 0.046). Nurses’ grit (mediator) directly affects career planning (dependent variable). This is represented by the path coefficient of 0.162 (*p* = 0.004). Organizational agility (independent variable) directly affects career planning (dependent variable). This is represented by the path coefficient of 0.143 (p 0.012). The indirect effect of organizational agility on career planning through nurses’ grit is calculated by multiplying the coefficients of both direct effects (0.115 * 0.162 = 0.709). This means that for each unit increase in organizational agility, we would expect a 0.709 unit increase in career planning through the mediating effect of nurses’ grit. This outcome can be explained by the way organizational agility supports nurses’ capacity to set and meet short- and long-term career goals, as well as how to develop professionals’ skills and experience through opportunities for training and development programs.

The study’s findings are in line with those of Yang et al. (2018) [10] and Yan et al. (2020) [12], who claimed that agile healthcare organizations support nurses’ career planning by giving them opportunities for growth and success in their careers. They can also incite nurses’ enthusiasm to boost morale and lower the likelihood of turnover. Additionally, organizational agility supports nurses in reaching their career planning objectives and encourages them to make stronger career plans and goals. Moreover, this result is in line with the findings of (El-Bahnasawy et al., 2021) [14]; (Wei et al., 2021) [15]; (Wang et al., 2022) [16] ; (Newsham, 2023) [17] who found that organizational agility gives healthcare organizations a competitive advantage by fostering the growth of gritty nurses who are interested in reaching their career planning objectives.

Similarly, nurses’ grit and career planning were favorably correlated with organizational agility (*r* = 0.166). Nurses’ grit (mediator) directly affects career planning (dependent variable). The reason for this outcome could be that organizational agility helps nurses set and meet both short- and long-term career goals, as well as how to get there. It also helps professionals grow in their knowledge and experience by offering opportunities for training and development programs such as professional development and continuous lifelong educational programs, setting up coaching or mentoring initiatives, and flexible career pathways to enhance nurses’ career planning and job satisfaction. These programs have produced highly gritty nurses who consistently show interest in their work, put in a lot of effort, and adjust their behavior as needed to reach career planning objectives.

This result is in line with the findings of (Park and Cho, 2019) [22] and (Lynda and Viola, 2019) [18], who claimed that organizational agility fosters nurses’ grit. This result also aligns with the findings of Yang and Kim (2022) [21], who stated that grit-filled healthcare professionals consistently keep an interest in goals, work hard, and adjust behaviors as necessary to meet career planning objectives. This outcome also aligns with the findings of Meyer et al. (2020) [19], who found that grittier nurses tend to keep going forward and use a variety of techniques to reach their career planning goals. Additionally, Cho and Kim (2022) [20], found that grittier nurses actively use the power of teamwork to persevere in achieving career goals. They also never stop developing their professional knowledge and skills and engaging in lifelong learning and nurses’ grit is a significant predictor of success and achievement of long-term career planning goals.

### Strengths and limitations

This study has approximations for returns. The cross-sectional methodology allowed for the simultaneous assessment of several factors in the population sample, resulting in precise data that was less vulnerable to potential biases found in case reports and case series. By using grit as a mediating element as perceived by nurses, the study broadens our understanding of the impact of organizational agility on career development, an area that receives little attention in the healthcare industry.

Nevertheless, there are certain limitations. First off, generalization is impossible because the study was limited to one hospital. Second, the sole dependent variable in this study looked at was the relationship between nurses’ opinions of career planning and their assessments of organizational agility by using grit as a mediating element. The effect of control variables is not clearly stated in the study, which may limit the interpretation of the results. Future research should consider the impact of control variables more explicitly to provide a comprehensive understanding of the findings. Future research can evaluate other factors that affect how well nurses can plan their jobs. Future studies can assess additional variables like work-life balance, chances for professional growth and continuing education, leadership support and organizational culture, the influence of nursing specializations or work environments on career trajectories, and more that influence how well nurses can plan their careers. Examining these extra factors can lead to a more thorough comprehension of the complex factors that go into nurses’ career planning and advancement in healthcare environments.

Additionally, the paper-based data entry and cleaning for the questionnaire required a lot of work. Finally, there is no indication that any of the study’s components are causally related. It was designed, after all, to look at the relationship between variables.

## Conclusion

Based on the findings of the current study, it could be concluded that there is a statistically high significant relationship between organizational agility and career planning. There is a statistically high significant relationship between organizational agility and grit. Also, there is a statistically high significant relationship between career planning and grit. Moreover, there is an impact of organizational agility on career development through grit as a mediating factor.

The findings highlight the worth of career planning and grit in fostering a positive work environment that promotes the development of nursing future and learning among nurses. Nursing is a career that stands out for its compassionate services and human-centeredness [51], thus career planning is the key to augmenting nurses’ career motivation [52]. Organizational agility training is advised as a part of daily operations for hospital and nursing managers to adapt quickly to opportunities and threats, achieve high standards of performance, and deliver quality services. Also, the provision of a high level of information and support to use their skills to plan for their career to achieve the task at hand and take ownership of their behavior. Concern for improving the quality of nurses` careers and being gritty nurses in terms of helping them to express their opinions and suggestions. Involvement of nurses in career decision-making, construction of career individualized plans, and programs to achieve organizational equilibrium.

Inclusive, this cross-sectional study increases our understanding of the relationships between organizational agility and career planning in nursing, highlighting the impact of organizational agility on career development through nurses’ grit as a mediating factor. This interconnectedness emphasizes the importance of these factors in promoting nurses’ future career well-being and accomplishment.

### Implications in nursing practice

The study’s conclusions also have significant ramifications for nursing practice and nurses’ daily work environments. Healthcare leaders and managers can put measures into place to better support nurses in their professional development and career trajectories by recognizing the impact of organizational agility and nurses’ grit on career planning. Nurse managers should work to promote an organizational culture that values flexibility and agility at the practice level. This could entail allowing nurses to take on new challenges and responsibilities, promoting cross-training and employment rotations, and establishing flexible work schedules. Nurse leaders can send a message to their nursing team that their career objectives are recognized and supported by exhibiting a commitment to organizational agility.

Healthcare organizations should also spend money on initiatives and materials that strengthen the fortitude and resiliency of nurses. This can entail giving nurses access to professional development programs, coaching, and mentorship so they can acquire the tenacity and drive required to meet their long-term career objectives. Acknowledging and honoring nurses who demonstrate exceptional grit can also encourage others to adopt a growth mindset and take charge of their career planning. In the end, work satisfaction, retention, and the general standard of patient care can all be significantly impacted by the combination of organizational agility and the development of nurses’ grit. Healthcare organizations can foster an environment that supports nurses’ professional and personal growth by giving priority to these elements, which will eventually benefit the patients and communities they serve.

### Implications for nursing education and policy

The study’s conclusions have several significant ramifications for healthcare policy and nursing education. The findings emphasize the value of developing grit and resilience in nursing students’ curriculum and early-career nurses from an educational perspective. Nurses will be more equipped to navigate the ever-changing healthcare landscape and plan their career paths through modules and activities included in nurse education programs. Additionally, healthcare organizations may create customized interventions to enhance nurses’ career progression and engagement including efforts to rethink jobs, mentorship programs, and chances for leadership development that empower nurses to take charge of their professional aspirations.

These results can direct the creation of workforce planning and retention plans for the nursing profession at the policy level. It is recommended that policymakers take into account the development of organizational agility in healthcare systems and allocate resources towards initiatives and programs that strengthen the resilience and career planning skills of nurses. By addressing the enduring issues of high turnover rates and nursing shortages, this all-encompassing strategy can eventually enhance patient outcomes and the long-term viability of the healthcare profession. Overall, this research provides a valuable foundation for bridging the gap between theory and practice in supporting nurses’ career development. By translating these insights into actionable educational and policy interventions, healthcare organizations can empower nurses to thrive in an ever-changing environment and contribute to the delivery of high-quality, patient-centered care.

### The impact and return on investment

To further strengthen the practical value of this study, the authors could incorporate an analysis of the potential impact and economic return of the recommended strategies. This could involve: Estimating the cost-savings or other financial benefits that may result from improved nurse retention, reduced turnover, and enhanced patient outcomes. Discussing the intangible, organizational benefits of investing in nurses’ career planning, such as increased job satisfaction, productivity, and quality of care. Providing a framework or model for healthcare organizations to evaluate the return on investment of implementing programs and initiatives aligned with the study’s findings.

## Data Availability

The datasets generated during and analyzed during the current study are available from the corresponding author upon reasonable request.
